# Association of Early Postoperative Pain Trajectories With Longer-term Pain Outcome After Primary Total Knee Arthroplasty

**DOI:** 10.1001/jamanetworkopen.2019.15105

**Published:** 2019-11-13

**Authors:** Jasvinder A. Singh, Celeste A. Lemay, Lisa Nobel, Wenyun Yang, Norman Weissman, Kenneth G. Saag, Jeroan Allison, Patricia D. Franklin

**Affiliations:** 1 Medicine Service, Birmingham Veterans Affairs Medical Center, Birmingham, Alabama; 2 Department of Medicine, The University of Alabama at Birmingham; 3 Division of Epidemiology, School of Public Health, The University of Alabama at Birmingham; 4 Department of Orthopedics and Physical Rehabilitation, University of Massachusetts Medical School, Worcester; 5 Department of Population and Quantitative Health Sciences, University of Massachusetts Medical School, Worcester

## Abstract

**Importance:**

Studies to date have not comprehensively examined pain experience after total knee arthroplasty (TKA). Discrete patterns of pain in this period might be associated with pain outcomes at 6 to 12 months after TKA.

**Objectives:**

To examine patterns of individual post-TKA pain trajectories and to assess their independent associations with longer-term pain outcome after TKA.

**Design, Setting, and Participants:**

This prospective cohort study combined data from a national US TKA cohort with ancillary pain severity data at 2 weeks and 8 weeks after the index TKA using a numeric rating scale. All participants received primary, unilateral TKA within the Function and Outcomes Research for Comparative Effectiveness in Total Joint Replacement (FORCE-TJR) national network of community sites in 22 states or at the lead site (University of Massachusetts Medical School). Participants had a date of surgery between May 1, 2013, and December 1, 2014. The data analysis was performed between January 13, 2015, and July 5, 2016.

**Exposures:**

Pain trajectories in the postoperative period (8 weeks).

**Main Outcomes and Measures:**

Index knee pain at 6 months after TKA using the Knee Injury and Osteoarthritis Outcome Score (KOOS) pain scale. Group-based trajectory methods examined the presence of pain trajectories in the postoperative period (8 weeks) and assessed whether trajectories were independently associated with longer-term pain (6 months).

**Results:**

The cohort included 659 patients who underwent primary TKA with complete data at 4 points (preoperative, 2 weeks, 8 weeks, and 26 weeks). Their mean (SD) age was 67.1 (8.0) years, 64.5% (425 of 659) were female, the mean (SD) body mass index (calculated as weight in kilograms divided by height in meters squared) was 30.77 (5.66), 94.5% (613 of 649) were white, and the mean (SD) preoperative 36-Item Short Form Health Survey physical component summary and mental component summary scores were 34.1 (8.2) and 53.8 (11.4), respectively. Two pain trajectory subgroups were identified at 8 weeks after TKA: patients who experienced fast pain relief in the first 8 weeks after TKA (fast pain responders, composing 72.4% [477 of 659] of the sample) and patients who did not (slow pain responders, composing 27.6% [182 of 659] of the sample). After adjusting for patient factors, the pain trajectory at 8 weeks after TKA was independently associated with the mean KOOS pain score at 6 months, with a between-trajectory difference of −11.3 (95% CI, −13.9 to −8.7).

**Conclusions and Relevance:**

The trajectory among slow pain responders at 8 weeks after surgery was independently associated with improved but greater persistent index knee pain at 6 months after TKA compared with that among fast pain responders. Early identification of patients with a trajectory of slow pain response at 8 weeks after TKA may offer an opportunity for interventions in the perioperative period to potentially improve the long-term pain outcomes after TKA.

## Key Points

**Question:**

Is knee pain recovery different between patient subgroups in the early postoperative period after total knee arthroplasty (TKA), and is it associated with intermediate-term TKA pain?

**Findings:**

In this cohort study of 659 patients who underwent primary TKA, 2 pain trajectories (fast pain response and slow pain response) were evident at 8 weeks after the procedure. These trajectories were independently associated with clinically significant differences in pain outcome at 6 months between the 2 pain trajectories.

**Meaning:**

Early identification of patients in the slow pain response trajectory at 8 weeks after TKA may offer an opportunity for interventions in the perioperative period to potentially improve the intermediate-term and long-term pain outcomes after TKA.

## Teaser

This cohort study examines patterns of individual pain trajectories after total knee arthroplasty (TKA) and assesses their independent associations with longer-term pain outcome after surgery.

## Introduction

Postoperative pain is commonly underestimated and undertreated.^1,2,3^ Undertreated postoperative pain not only leads to distress and poor patient satisfaction but also is associated with longer hospital stay, reluctance to engage in rehabilitation exercises, poorer health-related quality of life, and increased morbidity related to complications.^4,5,6,7,8^ Untreated postoperative pain is associated with persistent chronic pain after cardiac surgery.^6,9^

Total knee arthroplasty (TKA) is one of the most common elective surgical procedures performed in older patients to treat pain and functional limitation owing to refractory knee arthritis^10^ and is associated with optimal arthritis pain relief in most but not all patients. In the United States, 8% to 15% of patients who undergo TKA have residual moderate to severe index joint pain that persists 2 to 5 years after the procedure.^11,12^ A systematic review concluded that unfavorable long-term pain outcomes were seen in 10% to 34% of patients after knee arthroplasty.^13^ Few studies have examined intermediate-term postoperative pain and its association with long-term pain outcomes. In a single-center study^14^ of 116 patients, pain during the 1-year postoperative period generally improved, and 1-year pain status was associated with long-term pain outcomes. In 2 studies, researchers used preoperative and postoperative pain and function scores to assess pain outcome at 5 years after TKA^15^ or used preoperative scores to assess pain outcome at 3 months and 12 months after TKA^16^; these studies focused on preoperative pain and did not collect postoperative pain data (within 2 to 3 months of TKA).^15,16^ In addition, research to date is limited by single-center enrollment, small sample sizes with high dropout rates, and the lack of adjustment for common confounders (eg, race/ethnicity, body mass index [BMI; calculated as weight in kilograms divided by height in meters squared], and comorbidities).

To our knowledge, studies to date have not comprehensively examined pain experience in the post-TKA period or evaluated for the presence of discrete subgroups of individuals with different patterns of pain at 6 to 12 months after TKA. This factor is an important knowledge gap given (1) the co-occurrence of postoperative pain during post-TKA physical rehabilitation,^17,18^ (2) the treatment of early postoperative pain with opioids^19,20,21^ and the opioid epidemic in the United States,^22,23,24,25^ and (3) the significant prevalence of persistent pain after TKA.^11,12,13^ If early patterns of pain can be used to identify patients likely to experience prolonged pain, clinical treatment options could be developed and tailored to alter this outcome. The emerging tools for trajectory analysis shown to be associated with different care patterns and health care expenditures in patients with cancer^26^ offer the potential to provide new insights into patients’ pain experience and outcomes after TKA.

Our study objective was to examine whether discrete subgroups of patients undergoing TKA have different trajectories of pain in the period (8 weeks) after TKA in a nationally representative sample. We evaluated (1) whether the postoperative period pain trajectory is associated with the level of 6-month postarthroplasty pain outcome and (2) the patient characteristics associated with postarthroplasty pain trajectories.

## Methods

### Study Cohort and Survey

This research was an ancillary study of the Function and Outcomes Research for Comparative Effectiveness in Total Joint Replacement (FORCE-TJR) study,^27^ a nationally representative observational prospective cohort study that enrolled 28306 patients undergoing knee or hip arthroplasty (total or partial knee arthroplasty, total hip replacement, hip resurfacing, and knee or hip revision arthroplasty) across the United States, including more than 130 surgeons in academic and private centers. The FORCE-TJR study enrolled cases from April 11, 2011, to December 31, 2016. The study was approved by The University of Alabama at Birmingham and University of Massachusetts institutional review boards. Patient written informed consent was obtained before study enrollment and before any study activities were conducted. This study followed the Strengthening the Reporting of Observational Studies in Epidemiology (STROBE) reporting guideline.

The FORCE-TJR cohort was based on total knee replacement surgical procedures referred by a stratified sample of surgeons from across the United States; 75% of surgeons were from community-based practices. Operating room schedulers at each site referred patients scheduled for primary, unilateral TKA to the University of Massachusetts Medical School (UMMS) data coordinating center for enrollment each day. A centralized UMMS research coordinator reviewed participation by telephone with all patients. Almost 95% (12 585 of 13 283) of TKA patients who returned the signed informed consent form completed the preoperative assessment surveys, including demographics, preoperative pain and function (36-Item Short Form Health Survey [SF-36] and Knee Injury and Osteoarthritis Outcome Score [KOOS] pain scale), and medical and musculoskeletal risk factors according to the FORCE-TJR protocol. Specific enrollment and measures have been described previously.^27,28,29^ All FORCE-TJR participants repeated these measures at 6 months after total joint replacement. In addition to the comprehensive preoperative and outcome data collected by FORCE-TJR, patients who enrolled in the FORCE-TJR substudy during the ancillary study period at a subset of sites completed a 2-week and 8-week assessment of pain severity and pain treatment strategies.

All patients receiving TKA within the FORCE-TJR national network of community sites in 22 states or at the lead site (UMMS) were invited to enroll in the ancillary pain study, as well as the parent study.^27^ Exposures were pain trajectories in the postoperative period (8 weeks). For the ancillary pain study from 24 practice sites, patients who underwent primary, unilateral TKA also completed a pain assessment at 2 weeks and 8 weeks after the index TKA using a numeric rating scale (range, 0-10; higher scores indicate worse pain). Of the 12 585 FORCE-TJR study participants, 2209 (17.6%) of those undergoing primary total knee replacement were enrolled from May 12, 2011, to March 4, 2015. The dates of surgery were between May 1, 2013, and December 1, 2014. The dates of analysis were January 13, 2015, to July 5, 2016. Pain study sites included all community-based orthopedics offices covered by the University of Massachusetts institutional review board. The FORCE-TJR patients agreed to complete baseline, 6-month, and 12-month surveys, and the FORCE-TJR study staff supported collection of these data. Ancillary study resources supported a dedicated study coordinator to perform pain assessments at 2 weeks and 8 weeks for a subsample enrolled into the pain study. The consent forms were given to the patients at a preoperative visit, and surgeons requested each patient to participate in the registry. Next, coordinators called patients within 3 days to review the consent form to confirm participation. By reviewing operating room records, we verified that 79.4% (13 283 of 16 721) of people with TKA invited to join this ancillary study of the FORCE-TJR registry participated in the study. Patients received a reminder telephone call if the 2-week or 8-week survey was not returned within 2 weeks of the target date. Patients or other members of the public were not involved in the development of the study protocol.

### Pain Assessment Components

The 2-week and 8-week pain surveys contained the validated Revised American Pain Society Patient Outcome Questionnaire (APS-POQ-R),^30^ which includes pain severity (0-10 numeric rating scale; higher scores indicate worse pain),^31^ consequences of pain, common adverse effects of narcotics, and nonpharmacological strategies for managing postoperative pain. The pain numeric rating scale^31^ is valid, reliable, and feasible and has been used in multiple musculoskeletal conditions.^32,33,34,35,36^ We used the preoperative pain assessment along with 2-week and 8-week post-TKA pain severity to derive pain trajectories (detailed in the Statistical Analysis subsection).

### Long-term Pain Outcomes and Covariates

Early post-TKA pain trajectories (3 assessments, as well as preoperative pain severity) were the primary variables of interest. The main study outcome was index knee pain at 6 months after TKA as assessed with the KOOS pain scale as a continuous variable.^37,38^ The KOOS^39^ is a 42-item, participant-administered, validated, knee-specific questionnaire for osteoarthritis commonly used in clinical trials, registries, and patient follow-up and has been used in patients aged 13 to 79 years. The KOOS is valid and reliable, and the thresholds for minimal clinically important change have been defined.^40,41^ The KOOS covers 5 dimensions, including pain (9 items), knee-specific symptoms (7 items), activities of daily living (ADLs) function (17 items), sport and recreation function (5 items), and knee-related quality of life (4 items). Each item is rated by the patient on a 5-point Likert-type scale ranging from no problems to extreme problems. Each of the 5 scores is calculated as the sum of the items included. Scores are transformed to a scale of 0 to 100, with 0 representing extreme knee problems and 100 representing no knee problems. A difference of 8 to 10 points represents a clinically meaningful difference for the KOOS pain scale.^39,42^

Covariates included factors previously shown to be associated with longer-term pain outcome up to 6 months after TKA, including sex, age, BMI, race, medical comorbidity assessed using the modified Charlson Comorbidity Index^43^ (a validated measure), and quality of life assessed using the SF-36.^44,45^ The SF-36 physical component summary (PCS) and mental component summary (MCS) scores were calculated using standard algorithms; both are norm based, with a mean (SD) of 50 (10). Back pain was measured using the Oswestry Disability Index, with scores ranging from 0 to 50, categorized as no (0-4), mild (5-14), moderate (15-24), severe (25-34), or complete (35-50) disability.^46^

### Statistical Analysis

We performed all analyses using StataMP statistical software, version 13 (StataCorp LLC). We used a 2-sample Wilcoxon rank sum (Mann-Whitney) test (2 sided) for continuous variables and a Pearson χ^2^ test (1 sided) for categorical variables, with a significance level of .05 to examine whether there were any differences between the FORCE-TJR source cohort and the pain trajectory cohort. Descriptive statistics explored demographic characteristics, clinical variables, and pain scores before and after TKA. We used group-based trajectory models to assess trajectories of pain between pre-TKA and 8-week TKA scores.^47,48^ Specifically, we developed trajectories of the probability of having moderate to severe pain from before TKA to 8 weeks after TKA. We then examined factors associated with these trajectories and whether the trajectories could predict a pain outcome at 6 months. We used the trajectory program developed by Jones and Nagin.^49^

We assessed multicollinearity by examining the variance inflation factors for each of the variables in the model. A variable for which variance inflation factor values are greater than 10 may merit further investigation; the variance inflation factors were 1.08 or lower in our final linear regression model for 6-month outcome and in the logistic regression model for variables associated with trajectories. We examined the linearity assumption for variables that were continuous using the augmented partial residual plot, and no evidence of nonlinearity was found in our final linear regression model. The shape of the trajectories was forced to be linear owing to overfitting if quadratic or cubic terms were used with the limited number of time points that were available.

In sensitivity analyses, we evaluated the extent of missing surveys at 2 weeks and 8 weeks and whether any patterns of missingness altered trajectory assignment or outcome predictions. Missing pain values at 2 weeks or 8 weeks were imputed based on the predicted values from multilevel mixed-effects linear regression models.

#### Assessing the Trajectory Model

After basic exploratory data analysis has been performed, trajectory analysis requires assessment of (1) the number of trajectories, (2) the shape of each trajectory, and (3) the predictors of membership to each trajectory. We used several criteria to evaluate the group-based trajectory model to use. We assessed a priori 2 to 6 trajectory subgroups because models with more than 6 trajectory subgroups may be difficult to interpret clinically. We used the Bayesian information criterion to choose the optimal number of subgroups or trajectories.^48^ We also used the following criteria to assess model quality: (1) average posterior probability for membership in each trajectory of at least 0.70 and (2) nonoverlapping 95% CIs for the 2 trajectories.^48^ To avoid overfitting the data, we only examined linear trajectories for 3 points.^50^ We examined all combinations of linear trajectories and differing numbers of trajectories. Next, to create trajectory membership, all individuals were assigned to the trajectory to which they had the highest probability of belonging. This strategy was selected because our posterior probabilities of trajectory subgroup membership were high and using probabilistic membership in trajectories may not be useful in clinical practice. In other words, by using hard assignment to trajectories, we explicitly subgrouped patients according to pain scores.

#### Assessing the Factors Associated With Trajectory Membership

We used logistic regression to assess the factors associated with post-TKA pain trajectory membership, including patients who experienced pain relief in the postoperative period (fast pain responders) and patients who had less pain relief in the postoperative period (slow pain responders). Then all relevant factors associated with trajectory membership were screened for inclusion in the model. Those that were predictive (*P* < .10) were considered for inclusion in the final model. We calculated the area under the receiver operating characteristic curve to assess the ability of factors in the final model to explain the variability in the pain trajectory subgroups.^51^

#### Assessing the Predictive Ability of Trajectory Membership

We sought to assess whether the post-TKA pain trajectory was associated with pain severity at 6 months after TKA. We adjusted for preoperative covariates that are known factors associated with pain after TKA to assess the independent capability of trajectory subgroup assignment to predict pain. Model fit was examined using the Hosmer-Lemeshow test.^52^ We used adjusted *R^2^* to assess the ability of trajectory subgroups to explain the variability in the KOOS pain score at 6 months (ie, 26 weeks) after TKA.^51^ Based on resources for this ancillary study and the timing of its funding, we aimed to enroll more than 500 FORCE-TJR patients without using formal sample size calculations.

## Results

### Cohort Characteristics and Assessment of Nonresponse Bias

In total, 2209 patients undergoing TKA returned either 2-week or 8-week surveys (or both) during the enrollment period. We identified 659 patients with primary TKA from the FORCE-TJR cohort who had complete preoperative, 2-week, 8-week, and 26-week pain data and constituted the study cohort. Details are shown in the patient flowchart (eFigure in the Supplement). The mean (SD) age was 67.1 (8.0) years, 64.5% (425 of 659) were female, the mean (SD) BMI was 30.77 (5.66), 94.5% (613 of 649) were white, 68.8% (440 of 640) had an educational level of high school graduate or greater, and 66.9% (355 of 531) had a median annual household income exceeding $45 000 (Table 1). In addition, 63.8% (415 of 650) had a Charlson Comorbidity Index of 0, and 31.8% (203 of 638) had undergone a previous joint replacement surgery; the mean (SD) preoperative SF-36 PCS score and MCS score were 34.1 (8.2) and 53.8 (11.4), respectively (Table 1).

**Table 1.** Demographic and Preoperative Characteristics of the Study Cohort

Variable	No./Total No. (%) (N = 659)
Sex	
Male	234/659 (35.5)
Female	425/659 (64.5)
Age, mean (SD), y^a^	67.1 (8.0)
BMI^b^	
<25	90/640 (14.1)
25-29.9	214/640 (33.4)
30-34.9	202/640 (31.6)
35-39.9	97/640 (15.2)
≥40	37/640 (5.8)
Race	
White	613/649 (94.5)
Nonwhite	36/649 (5.5)
Marital status	
Married or living with someone as married	467/642 (72.7)
Widowed, separated, or divorced	142/642 (22.1)
Never married	33/642 (5.1)
Adults living in household, No.	
1	153/595 (25.7)
2	355/595 (59.7)
3	59/595 (9.9)
≥4	28/595 (4.7)
Educational level	
Less than high school graduate	183/640 (28.6)
High school graduate or greater	440/640 (68.8)
Missing	17/640 (2.7)
Insurance, plus secondary^c^	
Medicare	385/642 (60.0)
Private or HMO	230/642 (35.8)
All others^d^	27/642 (4.2)
Annual household income, median, $^e^	
≤45 000	176/531 (33.1)
>45 000	355/531 (66.9)
Charlson Comorbidity Index Score	
0	415/650 (63.8)
1	143/650 (22.0)
2	56/650 (8.6)
≥3	36/650 (5.5)
Previous joint replacement surgery	
No	435/638 (68.2)
Yes	203/638 (31.8)
Oswestry Disability Index low back pain	
None	282/647 (43.6)
Mild	191/647 (29.5)
Moderate	126/647 (19.5)
Severe	48/647 (7.4)
Nonsurgical joints with moderate to severe pain, No.	
0	475/650 (73.1)
1	142/650 (21.8)
2	22/650 (3.4)
3	11/650 (1.7)
Preoperative SF-36, mean (SD)	
PCS score	34.1 (8.2)
MCS score	53.8 (11.4)
Preoperative KOOS pain score for surgical knee, mean (SD)^f^	48.4 (17.7)
Preoperative KOOS pain score for surgical knee^f^	
None	3/659 (0.5)
Mild	65/659 (9.9)
Moderate	258/659 (39.2)
Severe	333/659 (50.5)
Preoperative KOOS pain score for nonsurgical knee, mean (SD)^f^	76.1 (23.3)
Preoperative KOOS pain score for nonsurgical knee^f^	
None	246/646 (38.1)
Mild	245/646 (37.9)
Moderate	118/646 (18.3)
Severe	37/646 (5.7)
Other preoperative KOOS scores for surgical knee, mean (SD)^f^	
Activities of daily living score	55.4 (18.5)
Symptom score	50.1 (20.1)
Sport score	18.7 (19.2)
Quality of life score	27.9 (17.7)

Abbreviations: BMI, body mass index (calculated as weight in kilograms divided by height in meters squared); HMO, health maintenance organization; KOOS, Knee Injury and Osteoarthritis Outcome Score; MCS, mental component summary; PCS, physical component summary; SF-36, 36-Item Short Form Health Survey.

^a^ In total, 221 of 659 (33.5%) were younger than 65 years, and 438 of 659 (66.5%) were 65 years or older.

^b^ The mean (SD) BMI was 30.8 (5.7).

^c^ Medicaid as secondary insurance was an option for 12 of 385 (3.1%) with Medicare, 2 of 278 (0.7%) with private/HMO, and 5 of 27 (18.5%) with all others.

^d^ All others include uninsured and self-pay.

^e^ Income exceeding $45 000 was chosen for categorization based on the national median income.

^f^ The KOOS pain scale ranges from 0 to 100 (0 represents extreme knee problems, and 100 represents no knee problems). A preoperative score of 50 to less than 70 indicates moderate pain, and 0 to less than 50 indicates severe pain. The KOOS items are rated by the patient on a 5-point Likert-type scale (range, 0-10; 0 represents no problems, and 10 represents extreme problems), with each of the 5 scores calculated as the sum of the items included; using a Likert-type 2-week and 8-week pain scale, a preoperative score of 5 to 6 indicates moderate pain, and 7 to 10 indicates severe pain.

In a comparison of patients in the ancillary pain study (patients responding to the 2-week and 8-week pain surveys) with all patients in the main FORCE-TJR cohort (the parent study), no meaningful clinical differences were identified despite some statistically significant differences in the preoperative variables (including sex, BMI, emotional health, and comorbidities). Within the pain survey cohort, 1655 of 3614 (45.8%) TJR patients (total knee replacement plus total hip replacement) completed both 2-week and 8-week surveys. No clinically meaningful differences in preoperative factors were found between pain survey respondents (at both 2 weeks and 8 weeks) and all FORCE-TJR participants with the exception of a difference in percentage of nonwhite patients (5.5% [36 of 659] in the pain cohort vs 10.3% [2145 of 20 783] in the FORCE-TJR cohort). eTable 1 in the Supplement summarizes the KOOS pain scores, and eTable 2 in the Supplement compares other patient characteristics.

### Factors Associated With Post-TKA Pain Trajectories

Two pain trajectory subgroups were identified at 8 weeks: fast pain relief in the first 8 weeks after TKA (fast pain response; 72.4% [477 of 659] of the sample) and no fast pain relief (slow pain response; 27.6% [182 of 659] of the sample) (Figure). The mean (SD) KOOS pain score at 6 months was 87.3 (13.4) in fast pain responders vs 74.5 (19.4) in slow pain responders.

**Figure.**
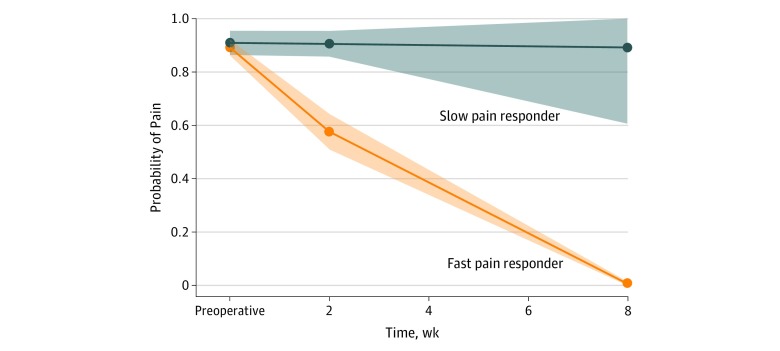
Pain Trajectory in Fast Pain Responders and Slow Pain Responders at 8 Weeks After Total Knee Arthroplasty Probability of moderate to severe pain is scaled 0 to 1 (lower indicates less probability, while higher indicates greater probability). The shaded areas are 95% CIs. Using the Knee Injury and Osteoarthritis Outcome Score (KOOS) pain scale (range, 0-100; 0 represents extreme knee problems, and 100 represents no knee problems), a preoperative score of 50 to less than 70 indicates moderate pain, and 0 to less than 50 indicates severe pain. The KOOS items are rated by the patient on a 5-point Likert-type scale (range, 0-10; 0 represents no problems, and 10 represents extreme problems), with each of the 5 scores calculated as the sum of the items included. Using the Likert-type 2-week and 8-week pain scale, a preoperative score of 5 to 6 indicates moderate pain, and 7 to 10 indicates severe pain.

Significant factors associated with the slow pain responder trajectory in univariate analyses were lower (worse) preoperative SF-36 MCS scores (51.5 vs 54.6; *P* = .002), more pain before surgery in the surgical knee (46.4 vs 49.2; *P* = .04), and worse preoperative KOOS ADL scores in the surgical knee (51.2 vs 57.0; *P* < .001) (eTable 3 in the Supplement). Sex, age, BMI, marital status, educational level, insurance status, Charlson Comorbidity Index, and previous joint replacement surgery were not significantly associated with the pain trajectory (eTable 3 in the Supplement). In multivariable-adjusted analyses, preoperative SF-36 MCS scores and preoperative KOOS ADL scores in the surgical knee were associated with the pain trajectory (Table 2). Each unit increase in SF-36 MCS and KOOS ADL score in surgical knee scores was associated with odds ratios of 0.98 (95% CI, 0.96-1.00; *P* = .02) and 0.97 (95% CI, 0.95-0.99; *P* = .007) for the slow pain responder trajectory, respectively.

**Table 2.** Multivariable-Adjusted Logistic Regression Model Assessing Pre–Total Knee Arthroplasty (TKA) Factors Associated With the Slow Pain Responder Trajectory^a^

Variable	Full Model^b^	
	OR (95% CI)	*P* Value
Preoperative SF-36 MCS score, with 1-unit increase	0.98 (0.96-1.00)	.02
Preoperative KOOS activities of daily living score for surgical knee, with 1-unit increase	0.97 (0.95-0.99)	.007
Sex		
Male	1 [Reference]	NA
Female	1.13 (0.73-1.73)	.58
Age, y		
<65	1 [Reference]	NA
≥65	0.97 (0.63-1.49)	.21
BMI		
<25		
25-29.9	1.97 (1.00-3.84)	.05
30-34.9	1.12 (0.56-2.24)	.74
≥35	1.43 (0.69-2.95)	.34
Race		
White	1 [Reference]	NA
Nonwhite	1.56 (0.70-3.49)	NA
Charlson Comorbidity Index Score		
0	1 [Reference]	NA
1	1.25 (0.79-2.00)	.34
2	1.20 (0.60-2.40)	.42
≥3	0.78 (0.32-1.91)	.35
Previous joint replacement surgery	0.82 (0.52-1.27)	.37
No	1 [Reference]	NA
Oswestry Disability Index low back pain		
None	1 [Reference]	NA
Mild	0.93 (0.58-1.49)	.76
Moderate	0.92 (0.53-1.58)	.76
Severe	0.87 (0.40-1.90)	.72
Nonsurgical joints with moderate to severe pain, No.		
0	1 [Reference]	NA
1	0.91 (0.54-1.51)	.70
2	1.17 (0.43-3.15)	.76
3	0.73 (0.17-3.07)	.67
Preoperative SF-36 PCS score, with 1-unit increase	1.00 (0.97-1.03)	.95
Preoperative KOOS pain score for surgical knee, with 1-unit increase	1.02 (1.00-1.04)	.06
Area under the receiver operating characteristic curve	0.64	NA

Abbreviations: BMI, body mass index (calculated as weight in kilograms divided by height in meters squared); KOOS, Knee Injury and Osteoarthritis Outcome Score; MCS, mental component summary; NA, not applicable; OR, odds ratio; PCS, physical component summary; SF-36, 36-Item Short Form Health Survey.

^a^ No evidence of multicollinearity was found because the variance inflation factors for each of the variables were 1.08 or lower (as a rule of thumb, a variable whose variance inflation factor values are greater than 10 may merit further investigation). Multivariable-adjusted logistic regression included the following factors: sex, age, BMI, race, preoperative Charlson Comorbidity Index score, previous joint replacement surgery, preoperative Oswestry Disability Index low back pain, preoperative number of nonsurgical joints with moderate to severe pain, preoperative SF-36 PCS score and MCS score, preoperative KOOS pain score for surgical knee, and KOOS activities of daily living score.

^b^ Only significant variables are listed in the table (significance level, *P* ≤ .05).

### Unadjusted and Multivariable-Adjusted Association of Pain Trajectories With 6-Month Pain

In univariate analyses, pain trajectories at 8 weeks were statistically significant and clinically meaningfully associated with the KOOS pain score at 6 months after TKA. Race, Charlson Comorbidity Index, and preoperative SF-36 MCS scores were also statistically significantly associated with the KOOS pain score at 6 months after TKA (Table 3). Nonwhite race was associated with a 9-point-lower KOOS pain score (75.4 vs 84.3, *P* = .01), Charlson Comorbidity Index of at least 3 with a 10-point-lower score compared with a score of 0 (74.7 vs 85.4, overall *P* < .001), and preoperative MCS score of at least 45 with a 10-point-higher score (85.7 vs 75.9, *P* < .001).

**Table 3.** Factors Associated With 6-Month Knee Injury and Osteoarthritis Outcome Score (KOOS) Pain Scores in Univariate Analyses^a^

Variable	KOOS Pain Score, Mean (SD)	*P* Value
Pain trajectory 8 wk after total knee arthroplasty		
Fast pain responders^b^	87.3 (13.4)	<.001
Slow pain responders^c^	74.5 (19.4)	
Sex		
Male	84.4 (17.4)	.16
Female	83.4 (15.7)	
Age, y		
<65	82.2 (18.8)	.43
≥65	84.5 (14.9)	
BMI		
<25	85.9 (13.6)	.08
25-29.9	82.9 (17.2)	
30-34.9	84.7 (16.4)	
35-39.9	80.9 (16.9)	
≥40	87.0 (14.3)	
Race		
White	84.3 (15.8)	.01
Nonwhite	75.4 (22.2)	
Annual household income, $		
≤45 000	82.3 (18.5)	.17
>45 000	85.3 (14.6)	
Charlson Comorbidity Index		
0	85.4 (15.5)	<.001
1	82.5 (17.3)	
2	80.1 (17.5)	
≥3	74.7 (16.3)	
Preoperative SF-36 MCS score		
<45	75.9 (19.3)	<.001
≥45	85.7 (14.8)	

Abbreviations: BMI, body mass index (calculated as weight in kilograms divided by height in meters squared); MCS, mental component summary; SF-36, 36-Item Short Form Health Survey.

^a^ Kruskal-Wallis test was used for the categorical variables in the table.

^b^ Patients who experienced pain relief in the immediate postoperative period are referred to as fast pain responders. The mean (SD) preoperative KOOS pain score for this subgroup was 49.2 (17.5), which was not different from the 46.4 (18.0) of slow pain responders. The improvement from preoperative to 6-month KOOS pain scores was 38.1 (19.2) in fast pain responders vs 28.1 (21.0) in slow pain responders.

^c^ Patients who experienced minimal pain relief in the immediate postoperative period are referred to as slow pain responders.

The pain trajectory at 8 weeks was significantly and clinically meaningfully associated with higher KOOS pain scores at 6 months after TKA in both the full model and reduced multivariable models. These results are summarized in Table 4. After adjusting for patient factors, the pain trajectory at 8 weeks after TKA was independently associated with the mean KOOS pain score at 6 months, with a between-trajectory difference of −11.3 (95% CI, −13.9 to −8.7; *P* < .001).

**Table 4.** Assessing the Association of 8-Week Pain Trajectory With 6-Month Knee Injury and Osteoarthritis Outcome Score (KOOS) Pain Scores^a^

Variable	Final Model 6-mo KOOS Pain Score (95% CI)^b^	*P* Value
Pain trajectory 8 wk after total knee arthroplasty		
Fast pain responders	1 [Reference]	NA
Slow pain responders	–11.3 (–13.9 to –8.7)	<.001
Race		
White	1 [Reference]	NA
Nonwhite	–6.6 (–11.6 to –1.5)	.01
Charlson Comorbidity Index Score		
0	1 [Reference]	NA
1	–1.3 (–4.1 to 1.5)	.36
2	–1.7 (–5.9 to 2.5)	.42
≥3	–6.8 (–11.8 to –1.8)	.008
Preoperative SF-36, with 1-unit increase		
PCS score	0.3 (0.1 to 0.4)	<.001
MCS score	0.3 (0.2 to 0.4)	<.001

Abbreviations: MCS, mental component summary; NA, not applicable; PCS, physical component summary; SF-36, 36-Item Short Form Health Survey.

^a^ The reduced 6-month model included pain trajectory, race, Charlson Comorbidity Index, and preoperative SF-36 PCS score and MCS score.

^b^ Because the *R^2^* value for the reduced model was the same as for the full model that included all the factors based on the principle of parsimony, reduced models were more desirable. No evidence of multicollinearity was found because the variance inflation factors for each of the variables were 1.08 or lower (a variable for which variance inflation factor values are greater than 10 may merit further investigation). The adjusted *R^2^* was 0.2

We performed sensitivity analyses by imputing pain scores for those missing them at 2 weeks or 8 weeks. The same variables that were significant in the main analyses were significant in the sensitivity analyses, with minimal attenuation of coefficients.

## Discussion

In this prospective cohort study of a representative US cohort, we combined data from the FORCE-TJR national cohort of primary TJR outcomes with ancillary pain survey data at 2 weeks and 8 weeks after TKA. We examined the patterns and characteristics of discrete posttrajectory subgroups in the first 8 weeks after surgery and how these trajectories predicted longer-term pain outcome after primary TKA. The 659 patients with preoperative, 2-week, and 8-week pain trajectory data were similar in patient profiles and comorbidities to the FORCE-TJR cohort. Two pain trajectory subgroups were identified at 8 weeks: two-thirds of patients who experienced fast pain relief in the postoperative period (fast pain responders) and one-third of patients who did not (slow pain responders). After adjusting for patient factors, the pain trajectory subgroup at 8 weeks after TKA was independently associated with the mean KOOS pain score at 6 months, indicating relevance to intermediate-term and long-term TKA outcomes.

Total knee arthroplasty is the most common and costly joint surgery, with approximately 700 000 TKA procedures being performed annually, making it a major public health burden.^53^ To our knowledge, the present work is the first study of a multisite, representative cohort of patients undergoing primary TKA in the United States that has examined the trajectory of pain after primary TKA and its association with pain outcomes at 6 months after surgery. Although previous studies^14,15,16,54^ examined preoperative pain (and in rare cases postoperative pain) and longer-term postoperative pain, to our knowledge, no systematic evaluation was done to assess pain trajectories and their association with longer-term outcomes (eg, patient pain experience). These retrospective studies also did not control for important confounders and were single-center studies, reducing confidence in the study findings and raising questions about generalizability. In the period after primary TKA, we found that 72.4% (477 of 659) of patients were fast pain responders and 27.6% (182 of 659) of patients were slow pain responders in the first 8 weeks. Evidence of the 2 pain trajectories existed as early as 8 weeks after the primary TKA and was possibly present even earlier. This observation was surprising and important because pain during this period includes postsurgical acute pain.^55,56^ Previous attempts at performing an analysis of pain trajectory were limited because they assessed preoperative factors only and did not investigate pain in the postoperative period.^15,16^ The findings of an association between preoperative variables and post-TKA pain in those single-center studies were similar to the results of other observational studies.^11,57,58,59,60,61^ Our study highlights the need to assess pain in patients during the period after primary TKA.

We also found that the postoperative pain trajectory was independently associated with longer-term post-TKA pain outcome. Specifically, although patients in the slow pain responder subgroup at 2 weeks and 8 weeks after surgery had significantly improved pain, they reported greater persistent index knee pain at 6 months after TKA compared with the fast pain responder subgroup. This finding has potential implications for patient care after primary TKA. Early identification of patients in the slow pain responder trajectory at 8 weeks after TKA (ie, for the subgroup continuing to experience moderate to severe pain) may offer an opportunity to target potentially modifiable factors for interventions in the perioperative period to possibly improve the long-term pain outcomes in patients who have undergone primary TKA. Persistent pain after TKA can contribute to long-term opioid use,^62,63^ which is a major public health crisis.^64,65^ Comprehensive pain management programs (eg, opioid prescribing guidelines^66^) and other behavioral interventions should be tested for this subgroup of patients in the postoperative period after TKA, with a goal to improve outcomes. Although the mean pain scores in the slow pain responder subgroup improved by 6 months, these patients did not reach the same status of the fast pain responder subgroup.

In the present study, preoperative physical health, general emotional and mental health, ADLs, and quality of life were associated with pain trajectory subgroups, whereas the other demographic or clinical characteristics were not. However, we did not assess immediate postoperative events and factors (ie, during the hospital stay and the first week after discharge to home) and thus are unable to provide additional insights regarding other postoperative variables amenable to intervention to improve outcomes. Future studies should examine whether the in-hospital, post-TKA period includes any modifiable factors associated with the 2-week to 8-week pain trajectory so that interventions targeting these factors may be developed and implemented.

The recent institution of bundled payments by the US Centers for Medicare & Medicaid Services (CMS) represents a major shift in the reimbursement of inpatient procedures, including TKA,^67^ but does not explicitly address patient-reported outcomes. According to this newly enacted policy, the CMS will pay for 90 days of care (ie, inpatient stay for TKA plus the first 90 days after discharge) rather than for each of the individual services to avoid fragmented care. This payment change by the CMS, which pays for most TKA procedures in the United States (totaling $7 billion in 2014 for knee and hip replacements^68^), will have major consequences on TKA reimbursement. A key change in adapting to the new payment system will be more explicit shared decision-making and a focus on improving patient-reported outcomes, as well as prevention of medical and surgical complications that lead to rehospitalization and increased cost of care.

Our study findings suggest that tailored pain treatment that targets the slow pain responders in the postoperative period should be tested for association with final pain outcomes. Taken together, the present study results and the new CMS payment initiative indicate the need for more research in this area and may ultimately result in improved patient pain outcomes and satisfaction. In addition, this study presents a new application of an emerging analytic method (trajectory analysis technique) to predict postoperative outcomes (using pain as an example). More research is needed in the arena of perioperative interventions, which have the potential to improve long-term post-TKA outcomes.^69,70^

### Strengths and Limitations

The study has notable strengths, including a national, pragmatic sample of contemporary patients undergoing TKA; use of a replicable, simple pain numeric rating scale at 2 weeks and 8 weeks; application of emerging trajectory tools; robustness of our study results; and a comprehensive assessment that included potential confounders and covariates in the analyses.

This study has limitations. First, although the patient profiles were comparable between the FORCE-TJR national cohort and our ancillary pain trajectory study sample, fewer nonwhite patients and patients with less comorbidity were included in the latter because of the nature of patients treated at the community sites that participated in the trajectory study. Second, this study did not collect pain data during the inpatient stay and the first week after TKA. This information may augment future post-TKA trajectory analyses. We included patients with complete data at 4 time points (preoperative, 2 weeks, 8 weeks, and 26 weeks) in these analyses. To ensure that patients with complete data were similar to the total FORCE-TJR cohort, we also performed sensitivity analyses, and the analytic subgroup did not meaningfully differ from the cohort.

## Conclusions

We identified 2 postoperative pain trajectories after primary TKA. The 2 trajectories were evident as early as 2 weeks after TKA and suggest that patients at risk of poor pain outcome may be identified after TKA. A simple pain numeric rating scale measure can be used after TKA in orthopedic practices to identify these pain trajectories. The wide availability of cell phones with texting ability and the use of electronic health records in orthopedic surgeon offices in the United States make this possible and feasible. We also found that the postoperative pain trajectory was independently associated with longer-term pain outcome at 6 months after TKA. Preoperative emotional and mental health status and ability to perform ADLs were associated with the pain trajectory and may guide tailored pain management. More research is needed to elucidate modifiable contributors to slow pain responder status in the postoperative period. Interventions targeting the postoperative pain trajectory and its correlates have the possibility to improve primary TKA outcomes.

## Notes

Supplement. **eFigure.** Patient Flow Chart **eTable 1.** Preoperative and Postoperative KOOS Pain Characteristics of FORCE-TJR Cohort, Patients at Eligible Site During Our Pain Study Enrollment, and Those Participating in Our Pain Study **eTable 2.** Comparison of Patient and Clinical Characteristics Between the FORCE-TJR Cohort and the Ancillary Pain Study Cohort **eTable 3.** Unadjusted Correlates of Pain Trajectories
