# Supplementary material for: Association of Early Postoperative Pain Trajectories With Longer-term Pain Outcome After Primary Total Knee Arthroplasty
Source: JAMA Netw Open. 2019 Nov 13;2(11):e1915105. doi: 10.1001/jamanetworkopen.2019.15105 (PMC6902788; doi:10.1001/jamanetworkopen.2019.15105)
Supplement: Supplement. — eFigure. Patient Flow Chart eTable 1. Preoperative and Postoperative KOOS Pain Characteristics of FORCE-TJR Cohort, Patients at Eligible Site During Our Pain Study Enrollment, and Those Participating in Our Pain Study eTable 2. Comparison of Patient and Clinical Characteristics Between the FORCE-TJR Cohort and the Ancillary Pain Study Cohort eTable 3. Unadjusted Correlates of Pain Trajectories [file jamanetwopen-2-e1915105-s001.pdf]

Supplementary Online Content

Singh JA, Lemay CA, Nobel L, et al. Association of early postoperative pain trajectories with longer-term pain outcome after primary total knee arthroplasty. *JAMA Netw Open*. 2019;2(11):e1915105. doi:10.1001/jamanetworkopen.2019.15105

eFigure. Patient Flow Chart

eTable 1. Preoperative and Postoperative KOOS Pain Characteristics of FORCE-TJR Cohort, Patients at Eligible Site During Our Pain Study Enrollment, and Those Participating in Our Pain Study

eTable 2. Comparison of Patient and Clinical Characteristics Between the FORCE-TJR Cohort and the Ancillary Pain Study Cohort

eTable 3. Unadjusted Correlates of Pain Trajectories

This supplementary material has been provided by the authors to give readers additional information about their work.

eFigure. Patient Flow Chart

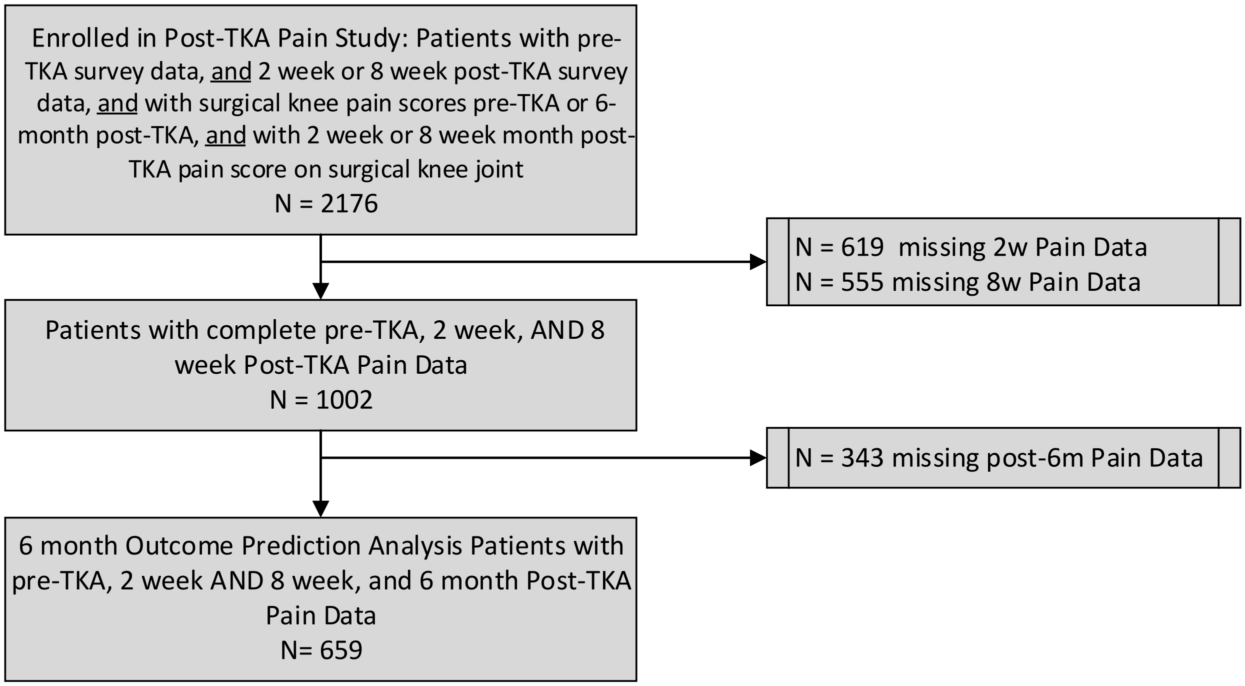

Figure 1 legend

Of the 2,209 people enrolled in the ancillary pain study who had pre-TKA survey data, and 2 week or 8 week post-TKA survey data, and surgical knee KOOS pain scores pre-TKA or 6-month post-TKA, only 2,176 provided data on pain score on surgical knee joint at 2 week or 8 week post-TKA follow-up.

© Singh JA et al. *JAMA Network Open*.

eTable 1. Preoperative and Postoperative KOOS Pain Characteristics of FORCE-TJR Cohort, Patients at Eligible Site During Our Pain Study Enrollment, and Those Participating in Our Pain Study

	N	Pre-operative		Post-TKA, 6 months		Change from Pre-operative to Post-TKA, 6 months	
		Mean	SD	Mean	SD	Mean	SD
FORCE-TJR cohort	11,354	46.0	17.8	81.7	16.9	34.3	20.9
Patients at Eligible sites	3,459	46.0	17.8	82.9	16.4	35.2	20.2
Patients participating in Pain study	2,209	47.2	17.8	83.6	16.4	35.2	20.3

Of the N = 12,585 that completed pre-op surveys in FTJR N=11,354 had KOOS pain scores both pre-operatively and at 6-month Post-TKA.

© Singh JA et al. *JAMA Network Open*.

eTable 2. Comparison of Patient and Clinical Characteristics Between the FORCE-TJR Cohort and the Ancillary Pain Study Cohort

	Ancillary study cohort **N=659 Mean [SD] or N (%)**	**FORCE-TJR cohort N=20,783† Mean [SD] or N (%)**
Gender		
**Male**	234 (35.5%)	8,214 (39.5%)
**Female**	425 (64.5%)	12,567 (60.5%)*
Mean Age [SD], in years	67.13 [8.0]	65.6 [9.9]
Race		
**White**	613 (93.0%)	18,280 (87.9%)
**Non-white**	36 (5.5%)‡	2,145 (10.3%)*
Marital Status		
**Married or Living with Someone as Married**	467 (70.9%)	13,618 (65.5%)
**Widowed, Separated or Divorced**	142 (21.5%)	4,879 (23.5%)
**Never Married**	33 (5.0%)‡	1,120 (5.6%)*
Education		
**High school graduate or less**	183 (27.8%)	5,898 (28.4%)
**Post high school graduate or more**	440 (66.8%)	13,235 (63.7%)
**Other**	17 (2.6%)‡	426 (2.0%)*
Annual Household Income		
**≤ $45,000**	176 (26.7%)	5,999 (28.9%)
**> $45,000**	355 (53.9%)‡	10,436 (50.2%)*
Charlson Comorbidity Count		
**0**	415 (63.0%)	11,569 (55.7%)
**1**	143 (21.7%)	4,153 (20.0%)
**2**	56 (8. 5%)	2,283 (11.0%)
**≥3**	36 (5.5%)‡	2,050 (9.9%)*
Mean Pre-operative KOOS pain score for surgical knee	48.4 [17.7]	44.2 [18.4]

KOOS, Knee Injury and Osteoarthritis Outcome Score; SD, standard deviation
† The FORCE-TJR Cohort of N=20,783 included both TKR and THR, of which the number of TKR were N=12,585

© Singh JA et al. *JAMA Network Open*.

‡ Missing/don't know for Ancillary study cohort: race, 10 (1%); marital status, 17 (3%); education level, 19 (3%); income, 128 (19.4%); Charlson comorbidity count, 9 (1.4%)
*** Missing/don't know for FORCE-TJR cohort: sex, 2 (<1%); race, 358 (1.7%); marital status, 1,166 (5.6%); education level, 1,224 (5.8%); income, 4,348 (20.9%); Charlson comorbidity count, 728 (3.5%)**

© Singh JA et al. *JAMA Network Open*.

eTable 3. Unadjusted Correlates of Pain Trajectories

	Pain Fast-responder* Pain Trajectory N=477<br>Mean [SD] or N (%)	Pain Slow-responder* Pain Trajectory N=182<br>Mean [SD] or N (%)	p-value
**Gender**			0.40
Male	174 (36%)	60 (33%)	
Female	303 (64%)	122 (67%)	
**Mean Age**	67.1 [8.0]	67.2 [8.0]	0.96
**Age in years**			0.72
<65	158 (33%)	63 (35%)	
≥65	319 (67%)	119 (65%)	
**Mean Body Mass Index (BMI), Kg/m^2**	30.9 (5.9)	30.5 (5.0)	0.68
**BMI categories, Kg/m^2**			0.06**
<25	69 (15%)	21 (12%)	
25-29.9	150 (32%)	64 (37%)	
30-34.9	153 (33%)	49 (28%)	
35-39.9	63 (13%)	34 (20%)	
≥40	32 (7%)	5 (3%)	
**Race**			0.10
White	451 (95%)	162 (92%)	
Non-white	22 (5%)	14 (8%)	
**Marital Status**			0.55
Married or Living with Someone as Married	344 (74%)	123 (70%)	
Widowed, Separated or Divorced	100 (21%)	42 (24%)	
Never Married	22 (5%)	11 (6%)	
**Adults Living in Household**			0.87
1	113 (26%)	40 (24%)	
2	254 (59%)	101 (61%)	
3	44 (10%)	15 (9%)	
≥4	19 (4%)	9 (5%)	

© Singh JA et al. *JAMA Network Open*.

**Education**			0.73
High school graduate or less	131 (28%)	52 (29%)	
Post high school graduate or more	321 (69%)	119 (67%)	
Other	11 (2%)	6 (3%)	
**Insurance**			0.87
Medicare (plus secondary)	278 (60%)	107 (61%)	
Private / HMO (plus secondary)	170 (36%)	60 (34%)	
All others	19 (4%)	8 (5%)	
**Annual Household Income**			0.08
≤ $45,000	123 (31%)	53 (39%)	
> $45,000	273 (69%)	82 (61%)	
**Charlson Comorbidity Count**			0.69
0	307 (65%)	108 (60%)	
1	99 (21%)	44 (25%)	
2	39 (8%)	17 (10%)	
≥3	26 (6%)	10 (6%)	
**Previous Joint Replacement surgery**			0.48
No	312 (67%)	123 (70%)	
Yes	151 (33%)	52 (30%)	
**Oswestry Low back pain**			0.53
None	207 (44%)	75 (42%)	
Mild	141 (30%)	50 (28%)	
Moderate	88 (19%)	38 (21%)	
Severe	31 (7%)	17 (9%)	
**Number of non-surgical joints with moderate/severe pain**			0.41
0	352 (74%)	123 (69%)	
1	100 (21%)	42 (24%)	
2	13 (3%)	9 (5%)	
3	8 (2%)	3 (2%)	

© Singh JA et al. *JAMA Network Open*.

**Mean Pre-operative SF 36 Mental Component Summary (MCS) score**	54.6 [11.1]	51.5 [12.0]	0.002
**Mean Pre-operative SF 36 Physical Component Summary (PCS) score**	34.5 [8.2]	33.1 [8.0]	0.06
**Mean Pre-operative KOOS pain score for surgical knee**	49.2 [17.5]	46.4 [18.0]	0.037
**Pre-operative KOOS pain score for surgical knee*** **			0.07
None	1 (0%)	2 (1%)	
Mild	50 (10%)	15 (8%)	
Moderate	197 (41%)	61 (34%)	
Severe	229 (48%)	104 (57%)	
**Mean Pre-operative KOOS pain score for non-surgical knee**	76.8 [23.3]	74.1 [23.3]	0.12
**Pre-operative KOOS pain score for non-surgical knee*** **			0.54
None	184 (39%)	62 (35%)	
Mild	180 (38%)	65 (37%)	
Moderate	80 (17%)	38 (22%)	
Severe	26 (6%)	11 (6%)	
**Mean KOOS Other Scores for surgical knee**			
Pre-operative KOOS Activities of Daily Living score	57.0 [18.4]	51.2 [17.9]	0.0001
Pre-operative KOOS Symptom score	50.0 [20.0]	50.2 [20.4]	0.97
Pre-operative KOOS Sport score	19.4 [19.4]	17.0 [18.7]	0.09
Pre-operative KOOS Quality of Life score	28.0 [16.9]	27.6 [19.6]	0.48

Fisher exact test or chi-square test was used for categorical variables, and rank sum test was used for continuous variables.
* Patients who experienced pain relief in the immediate post-operative period are referred to as Pain-responder
Patients who experience minimal pain relief in the immediate post-operative period are referred to as Pain slow-responders
** Due to small number cells (<5), Fisher's exact test was used
***KOOS pain subscale: 100, no pain; 70-100, mild pain; 50-<70, moderate pain; 0-<50, severe pain,

© Singh JA et al. *JAMA Network Open*.
